# Fibroblast activation in sarcoidosis as assessed by ^68^Ga-FAPI (fibroblast activation protein inhibitor)-46 PET/CT

**DOI:** 10.1093/qjmed/hcae067

**Published:** 2024-04-12

**Authors:** S O’Brien, K Musameh, M El Obeid, T Butler, M Armstrong, Z Cunningham, A Atzinger, T Kuwert, P D Mitchell, S C Donnelly

**Affiliations:** Department of Clinical Medicine, School of Medicine, Trinity College Dublin, Dublin, Ireland; Department of Clinical Medicine, School of Medicine, Trinity College Dublin, Dublin, Ireland; Department of Clinical Medicine, School of Medicine, Trinity College Dublin, Dublin, Ireland; Department of Clinical Medicine, School of Medicine, Trinity College Dublin, Dublin, Ireland; Department of Clinical Medicine, School of Medicine, Trinity College Dublin, Dublin, Ireland; Department of Clinical Medicine, School of Medicine, Trinity College Dublin, Dublin, Ireland; Department of Nuclear Medicine, Friedrich-Alexander University Erlangen and University Hospital Erlangen, Erlangen, Germany; Department of Nuclear Medicine, Friedrich-Alexander University Erlangen and University Hospital Erlangen, Erlangen, Germany; Department of Clinical Medicine, School of Medicine, Trinity College Dublin, Dublin, Ireland; Department of Clinical Medicine, School of Medicine, Trinity College Dublin, Dublin, Ireland

Learning point for clinicians ^68^Ga-FAPI-46-PET/CT is a promising imaging modality which displays activated fibroblasts. This may have great potential for evaluating fibroblast activity in conditions such as sarcoidosis and to identify patients at risk of progressive pulmonary fibrosis at an earlier stage. ^68^Ga-FAPI-46-PET/CT could also play a role in aiding therapeutic decision making and monitoring therapeutic response in patients with pulmonary fibrosis.

## Introduction

^68^Ga-FAPI-46-PET/CT is a promising imaging modality which highlights activated fibroblasts that are involved in fibrotic processes such as in interstitial lung diseases.^1^ It has been previously demonstrated that ^68^Ga-FAPI-46-PET/CT can discriminate between inflammation and fibrosis in other diseases.^2^ We report on the case of ^68^Ga-FAPI-46-PET/CT detection of fibroblast activity in active pulmonary sarcoidosis.

## The case

A 35 year old male was admitted with a 3 month history of dyspnea and dry cough. He had no comorbidities and was on no medication. CT thorax demonstrated diffuse pulmonary parenchymal groundglass change and nodularity with associated bulky mediastinal adenopathy. Subsequent endobronchial ultrasound and transbronchial node aspirate of mediastinal lymph nodes demonstrated non-caseating granulomas and the patient was diagnosed with pulmonary sarcoidosis. Bronchoalveolar lavage white cell differential demonstrated a lymphocyte count of 18%, CD4: CD8 ratio of 5:1, supportive of a diagnosis of sarcoidosis. Mycobacteriological studies were negative. Pulmonary function testing was performed: FEV1 3.07 L (82%), FVC 3.65 L (82%), FEV1/FVC 84%, TLCO 67%.

The patient underwent ^68^Ga-FAPI-46-PET/CT to evaluate for fibroblast activity. Radiotracer Gallium-68 FAPI was injected intravenously. 45 min post injection, we acquired PET scans of the whole body from head to toe craniocaudally.

The CT acquisition was performed with 100 kV and 40 reference mAs (milliampere-seconds).

**Figure 1.**
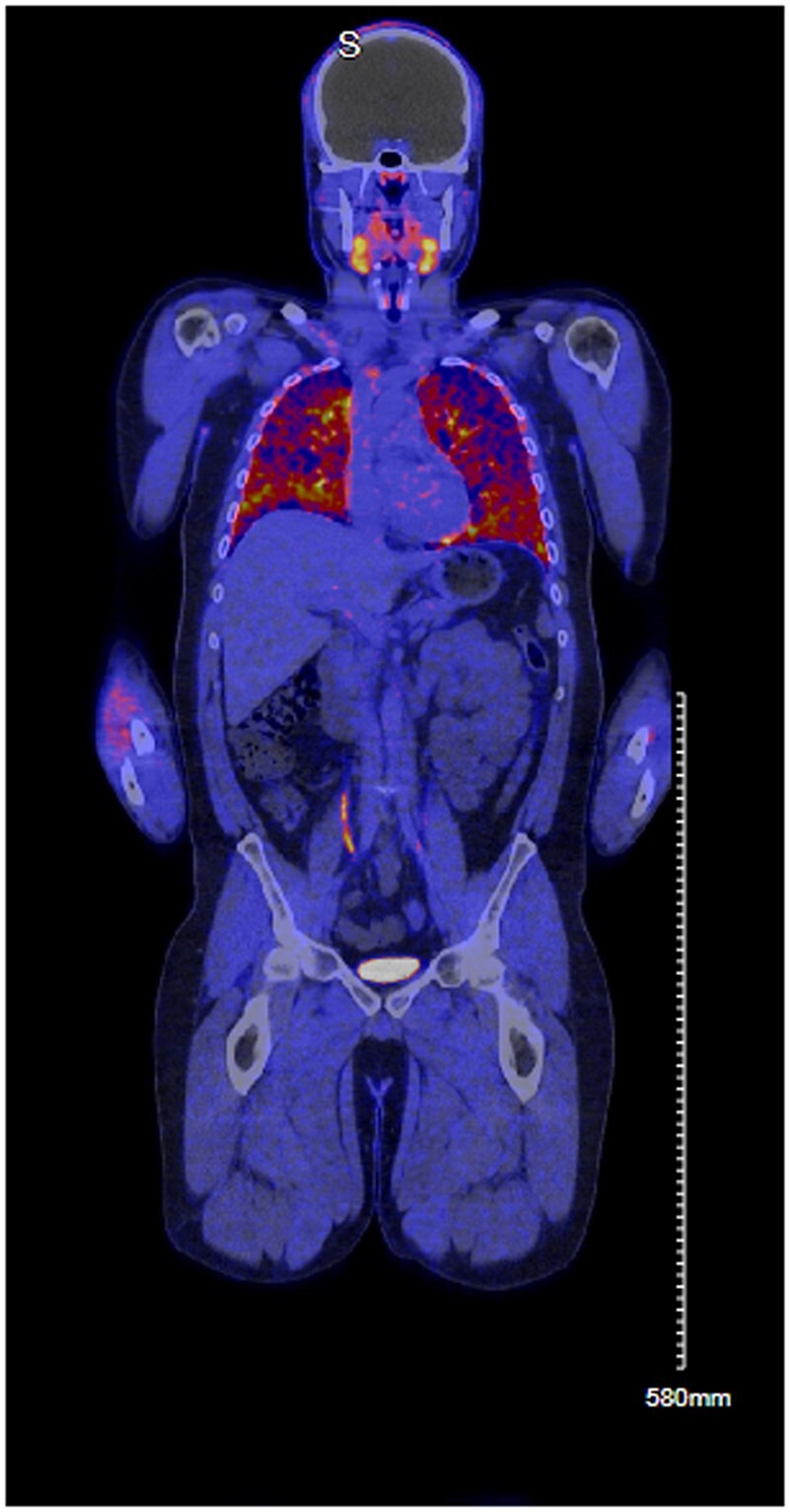
68Ga-FAPI-46-PET/CT demonstrated intensive FAPI uptake in the lung parenchyma (as depicted using the Hot Metal Colour Scale with blue areas signifying low uptake and orange and yellow areas signifying higher uptake).

This ^68^Ga-FAPI-46-PET/CT demonstrated intensive FAPI uptake in the lung parenchyma throughout (Figure 1). Mediastinal lymphadenopathy without significant FAPI uptake, was also noted. He was subsequently commenced on a tapering course of corticosteroids starting at 20 mg of prednisolone with a good clinical response.

## Discussion

Progressive fibrosis is a major cause of morbidity and mortality in pulmonary sarcoidosis with respiratory failure the leading cause of sarcoidosis-related death.^3^ There is evidence that fibroblast activation on ^68^Ga-FAPI-46-PET/CT correlates with fibrotic activity and disease progression in patients with fibrotic lung disease.^4^ This case demonstrates that molecular imaging can highlight activated fibroblasts in the lung parenchyma.

It is estimated that 15% of patients with sarcoid associated lung disease will develop progressive pulmonary fibrosis.^5^ We do not possess accurate methods of predicting progressive pulmonary fibrosis in sarcoidosis at present. While imaging modalities such as CT and PET CT can accurately detect the fibrotic burden and inflammatory burden, respectively, ^68^Ga-FAPI-46-PET/CT offers clinicians a non-invasive method to visualize and quantify fibroblast expression in the lung and has great potential value for early identification of patients at risk of progressive pulmonary fibrosis in sarcoidosis. Treatments targeting fibrotic pathways may prove to be beneficial for those patients with FAPI uptake on imaging.

## Conflict of interest

None declared. 
